# *In vitro* evaluation of dual carbapenem combinations against carbapenemase-producing *Pseudomonas aeruginosa*

**DOI:** 10.1093/jac/dkae445

**Published:** 2024-12-10

**Authors:** Ying Zhu, Jacqueline Findlay, Maxime Bouvier, Qiwen Yang, Yingchun Xu, Patrice Nordmann

**Affiliations:** Department of Clinical Laboratory, State Key Laboratory of Complex Severe and Rare Diseases, Peking Union Medical College Hospital, Chinese Academy of Medical Sciences, Beijing, China; Medical and Molecular Microbiology, Faculty of Science and Medicine, University of Fribourg, Chemin du Musée 18, Fribourg CH-1700, Switzerland; Graduate School, Peking Union Medical College, Chinese Academy of Medical Sciences, Beijing, China; Medical and Molecular Microbiology, Faculty of Science and Medicine, University of Fribourg, Chemin du Musée 18, Fribourg CH-1700, Switzerland; Swiss National Reference Center for Emerging Antibiotic Resistance (NARA), University of Fribourg, Fribourg, Switzerland; Medical and Molecular Microbiology, Faculty of Science and Medicine, University of Fribourg, Chemin du Musée 18, Fribourg CH-1700, Switzerland; Swiss National Reference Center for Emerging Antibiotic Resistance (NARA), University of Fribourg, Fribourg, Switzerland; Department of Clinical Laboratory, State Key Laboratory of Complex Severe and Rare Diseases, Peking Union Medical College Hospital, Chinese Academy of Medical Sciences, Beijing, China; Department of Clinical Laboratory, State Key Laboratory of Complex Severe and Rare Diseases, Peking Union Medical College Hospital, Chinese Academy of Medical Sciences, Beijing, China; Medical and Molecular Microbiology, Faculty of Science and Medicine, University of Fribourg, Chemin du Musée 18, Fribourg CH-1700, Switzerland; Swiss National Reference Center for Emerging Antibiotic Resistance (NARA), University of Fribourg, Fribourg, Switzerland

Lucía *et al*.^1^ recently evaluated the newly developed β-lactam and β-lactam/β-lactamase inhibitor combinations against β-lactam-resistant *Pseudomonas aeruginosa*. This has highlighted the need for new treatments for infections caused by *P. aeruginosa*, especially carbapenem-resistant *P. aeruginosa* (CRPA), which the World Health Organization has listed as a high-priority pathogen (https://www.who.int/publications/i/item/9789240093461) in 2024. CRPA infections often lead to high mortality in intensive care units^2^ and can be difficult to treat due to both intrinsic and acquired resistance mechanisms, resulting in limited antibiotic options.^1,3,4^ Mutations in outer membrane porin OprD and the acquisition of metallo-β-lactamases (MBLs) (mostly NDM-1, VIM-2 and IMP-1) or class A enzymes (KPC-2) are the most common mechanisms of resistance to carbapenems in *P. aeruginosa*.^2^ Different antibiotic combinations and very few novel antibiotics are new strategies to counter the serious problem of antimicrobial resistance in *P. aeruginosa,* such as ceftazidime-avibactam, ceftolozane-tazobactam and cefiderocol.^1^

Several studies have suggested dual carbapenem combinations for *Acinetobacter baumannii* and Enterobacterales as a possibility for treating CR organisms through *in vitro* experiments.^5–8^ Oliva *et al.*^8^ applied meropenem plus ertapenem administration in three patients infected with CR *Klebsiella pneumoniae* and achieved improved effectiveness. Mackay *et al.*^9^ has reported dual/multi-carbapenem treatments improved efficacy against infections by VEB-1 and VIM-10-carrying *P. aeruginosa* compared with monotherapies. However, the efficacy of carbapenem combinations against the main types of carbapenemase-producing *P. aeruginosa* has not been tested and the larva model is debatable.^9^ Therefore, this study aimed to evaluate the *in vitro* activity of dual carbapenem combinations against CRPA isolates carrying the most widespread carbapenemases. Imipenem, meropenem and ertapenem were evaluated since they are widely used in clinics. Although *P. aeruginosa* is naturally resistant to ertapenem, it was also tested given that it can saturate the targets (penicillin-binding proteins) and then confer a potential increase in the bactericidal activity of another carbapenem.^6,9^

Twenty non-clonally related clinical *P. aeruginosa* isolates and isogenic PAO1 strains with/without a functional OprD and harbouring different carbapenemases (AIM-1, KPC-2, IMP-1, NDM-1, SPM-1 and VIM-2) were tested with checkerboard synergy testing.^7^ Briefly, the combinations of two different serial-diluted carbapenems were tested for each strain. The clinical strains originated from the Swiss National Reference Center for Emerging Antibiotic Resistance and from the lab personal collection and PAO1 reference *P. aeruginosa* strain with disrupted *oprD* was obtained from PAO1 transposon mutant library (https://sites.google.com/uw.edu/salipante-lab). All isolates were resistant to imipenem and meropenem (Table 1), according to CLSI guidelines,^10^ and MICs of ertapenem in were, as expected, all ≥64 mg/L. The fractional inhibitory concentration index (FICI) was calculated as FIC of drug A plus FIC of drug B, as FIC of a drug refers to  the MIC of drug A or B in combination divided by the MIC of the drug alone.^7^ Synergy was considered when FICI≤0.5 while no interaction was considered when 4>FICI>0.5 and antagonism was considered when FICI>4.^7^

**Table 1.** Evaluation of the efficacy of dual carbapenem combinations against carbapenem-resistant *Pseudomonas aeruginosa*

	Name	Carbapenemase	OprD	MIC (g/mL)			FICI		
				IPM	MEM	ETP	IPM/MEM	IPM/ETP	MEM/ETP
1	PAO1-AIM-1	AIM-1	WT	128	256	1024	0.56	**0**.**50**	0.75
2	PAO1-TnoprD-AIM-1	AIM-1	truncated	256	512	1024	0.63	0.75	0.75
3	PAO1-IMP-1	IMP-1	WT	64	256	1024	**0**.**38**	**0**.**50**	0.75
4	PAO1-TnoprD-IMP-1	IMP-1	truncated	256	512	>1024	0.56	**0**.**50**	0.63
5	PAO1-KPC-2	KPC-2	WT	128	256	1024	**0**.**50**	**0**.**38**	0.75
6	PAO1-TnoprD-KPC-2	KPC-2	truncated	256	512	>1024	0.63	**0**.**50**	0.56
7	PAO1-NDM-1	NDM-1	WT	64	128	512	**0**.**50**	0.75	0.75
8	PAO1-TnoprD-NDM-1	NDM-1	truncated	256	256	1024	**0**.**28**	0.75	0.63
9	PAO1-SPM-1	SPM-1	WT	8	64	512	1.50	**0**.**50**	0.63
10	PAO1-TnoprD-SPM-1	SPM-1	truncated	64	128	512	0.75	0.63	0.56
11	PAO1-VIM-2	VIM-2	WT	32	32	128	0.56	**0**.**50**	1.50
12	PAO1-TnoprD-VIM-2	VIM-2	truncated	128	64	512	1.00	0.75	0.75
13	A101	GES-5	WT	32	128	256	**0**.**38**	**0**.**38**	1.00
14	A102	IMP-1	WT	32	128	1024	0.63	**0**.**38**	1.00
15	A103	IMP-1	truncated	128	512	>1024	0.63	**0**.**50**	0.63
16	A104	IMP-7	truncated	64	256	512	**0**.**50**	0.75	0.75
17	A105	IMP-13	WT	32	64	512	1.50	0.75	1.76
18	A106	KPC-2	NA	128	128	512	**0**.**38**	**0**.**38**	0.63
19	A107	KPC-2	NA	256	512	>1024	**0**.**31**	**0**.**38**	0.63
20	A108	NDM-1	truncated	256	512	1024	0.75	0.63	**0**.**50**
21	A109	NDM-1	deleted	256	>512	>1024	0.63	0.75	0.63
22	A110	NDM-1	WT	128	256	1024	**0**.**28**	**0**.**50**	1.00
23	A111	NDM-1	WT	256	512	>1024	**0**.**50**	0.53	0.52
24	A112	SPM-1	truncated	256	512	1024	0.56	0.63	**0**.**50**
25	A113	VIM-1	truncated	>256	512	1024	**0**.**50**	0.63	0.56
26	A114	VIM-2	truncated	256	128	256	0.56	0.75	1.00
27	A115	VIM-2	WT	16	8	64	0.75	**0**.**38**	**0**.**50**
28	A116	VIM-2	truncated	256	64	256	0.63	0.63	0.75
29	A117	VIM-2	truncated	256	128	512	**0**.**50**	0.63	0.75
30	A118	VIM-4	WT	>256	>512	1024	0.53	0.63	**0**.**31**
31	A119	VIM-4	truncated	>256	256	1024	0.75	0.56	0.75
32	A120	NDM-1, VIM-2	truncated	256	256	512	0.63	1.00	0.56

The FICI with synergy efficacy is shown in bold.

MIC, minimum inhibition concentration; FICI, fractional inhibitory concentration index; IPM, imipenem; MEM, meropenem; ETP, ertapenem; WT, wild type; NA, not available.

Within the isogenic strains, 50% (7/12) showed synergy with the imipenem/ertapenem combination, including five wild-type PAO1 and two with truncated OprD. Synergy for imipenem/meropenem combination was found in 33.3% (4/12) of transformants, mainly in wild-type strains (75%, 3/4). No transformants showed synergy with the meropenem/ertapenem combination.

The carbapenemase genes and OprD status of clinical isolates had previously been determined by whole-genome sequencing. The clinical strains consisted of producers of various carbapenemases with different *oprD* formations. The imipenem/meropenem combination exhibited a synergistic effect in 40% (8/20) of clinical isolates, imipenem/ertapenem in 35% (7/20) while meropenem/ertapenem only in 25% (5/20). No antagonism was observed in any combination. The isolates where a synergistic effect was observed produced various carbapenemases. The isolates with wild-type *oprD* exhibited greater synergy than those with a non-functional OprD in all combinations (imipenem/meropenem: 42.9% versus 27.3%; imipenem/ertapenem: 57.1% versus 9.1%; meropenem/ertapenem: 28.6% versus 18.2%).

The rate of *in vitro* synergy effect observed from imipenem/meropenem combination and imipenem/ertapenem combination in CRPA was similar to that in CR *K. pneumoniae* or CR *A. baumannii.*^6,7^ The rate of meropenem/ertapenem combination in CRPA (25%) was higher than that of CR *K. pneumoniae* (0%). Interestingly, clones with OprD truncated exhibited less synergy activity to dual carbapenem combinations treatment, likely due to the main role of OprD as a porin for carbapenem to enter *P. aeruginosa* and the truncation of OprD greatly affects the penetration of carbapenems.^3^

Our study suggests dual carbapenem combinations have antimicrobial effect to CRPA, particularly for not only MBL-producers but also class A carbapenemase-producers. Given the limitation of choices for treating CRPA infections, these dual carbapenem combinations could provide more alternatives. Notably, since *P. aeruginosa* is naturally resistant to ertapenem, the synergistic combination with ertapenem would be beneficial for treating CRPA as there is no concern about acquired resistance. Further research in animal infectious models and clinical trials is needed to explore the *in vivo* efficacy mechanisms of synergy effect from these combinations.
